# Mitochondrial morphology provides a mechanism for energy buffering at synapses

**DOI:** 10.1038/s41598-019-54159-1

**Published:** 2019-12-04

**Authors:** Guadalupe C. Garcia, Thomas M. Bartol, Sébastien Phan, Eric A. Bushong, Guy Perkins, Terrence J. Sejnowski, Mark H. Ellisman, Alexander Skupin

**Affiliations:** 1 Luxembourg Centre for Systems Biomedicine, University of Luxembourg, Belvaux, L-4367 Luxembourg; 2 Computational Neurobiology Laboratory, Salk Institute for Biological Studies, La Jolla, CA 92037 USA; 3 National Center for Microscopy and Imaging Research, Center for Research in Biological Systems, University of California, San Diego, La Jolla, CA 92093 USA

**Keywords:** Biophysics, Cell biology, Computational biology and bioinformatics, Neuroscience, Systems biology

## Abstract

Mitochondria as the main energy suppliers of eukaryotic cells are highly dynamic organelles that fuse, divide and are transported along the cytoskeleton to ensure cellular energy homeostasis. While these processes are well established, substantial evidence indicates that the internal structure is also highly variable in dependence on metabolic conditions. However, a quantitative mechanistic understanding of how mitochondrial morphology affects energetic states is still elusive. To address this question, we here present an agent-based multiscale model that integrates three-dimensional morphologies from electron microscopy tomography with the molecular dynamics of the main ATP producing components. We apply our modeling approach to mitochondria at the synapse which is the largest energy consumer within the brain. Interestingly, comparing the spatiotemporal simulations with a corresponding space-independent approach, we find minor spatial effects when the system relaxes toward equilibrium but a qualitative difference in fluctuating environments. These results suggest that internal mitochondrial morphology is not only optimized for ATP production but also provides a mechanism for energy buffering and may represent a mechanism for cellular robustness.

## Introduction

Mitochondria are subcellular organelles well-known as the powerhouses of eukaryotic cells where metabolic substrates are converted to adenine triphosphate (ATP), the main energy substrate of life^1^. Dependent on their physiological context, mitochondria exhibit diverse phenotypes and their dysfunction is linked to diverse metabolic diseases and also to cancer^2^, diabetes^3^ and neurodegeneration^4^. The specific energetic needs of the brain and in particular of synaptic transmission is accompanied by a distinct mitochondrial phenotype on the molecular as well as on the morphological level^5^. Impairment of presynaptic homeostasis caused by mitochondrial dysfunction is believed to contribute significantly to neurodegeneration^5^, and compromised mitochondrial morphology is correlated with insufficient ATP production^6^. Hence, understanding the interplay between molecular and morphological features of mitochondria may provide new insights into brain energy homeostasis and mechanisms of neurodegeneration.

**Figure 1:**
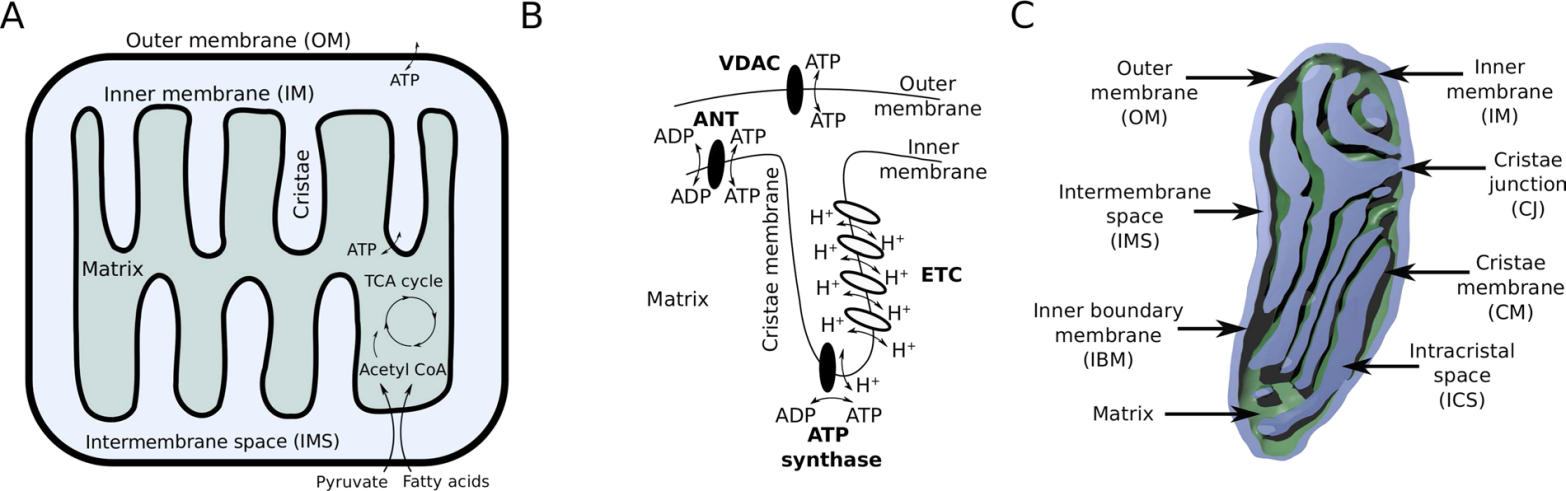
Mitochondrial morphology and function. (**A**) Functional morphology of mitochondria. The outer membrane separates the mitochondrion from the cytosol and the inner membrane encloses the matrix where the enzymes of TCA cycle are located which use pyruvate and fatty acids as substrates to drive ATP production by oxidative phosphorylation. The two membranes are separated by the intermembrane and intracristal space, formed by invaginations of the inner membrane. (**B**) Schematic representation of the cristae organization and protein localization involved in ATP production. The ETC generates a chemo-electrical gradient $\Delta \Phi$ across the inner membrane by pumping protons (H${}^{+}$) into the cristae. ATP-synthases utilize this gradient to drive protons back in the matrix for phosphorylation of ADP to ATP. From the matrix, ATP is transported by the ANT into the intermembrane space and cross the OM at VDACs to the cytosol. (Adenosine handling proteins are shown in black and other mitochondrial proteins generating the electrochemical gradient are shown in white). (**C**) Detailed physiological annotation of a reconstructed mitochondrion. The outer membrane and the inner membrane are separated by the intermembrane space. The inner membrane builds the matrix of the mitochondrion (green) and can be topologically divided into the inner boundary membrane and the cristal membrane which merge at the tubular cristae junctions where the intracristal space is connected to the intermembrane space.

Mitochondria exhibit a specialized morphology that implements an efficient framework for oxidative phosphorylation (oxphos) of adenosine diphosphate (ADP) to ATP. The mitochondrial structure is characterized by two membranes, with one membrane surrounding the other where the outer membrane (OM) separates the mitochondrion from the cytosol and the inner membrane (IM) defines the matrix (Fig. 1A). The core of the oxphos machinery is an electro-chemical gradient $\Delta \Phi$ across the IM that is driven by the tricarboxylic acid (TCA) cycle in the matrix^7^. The intermediates of the TCA cycle trigger the electron transport chain (ETC) to pump protons out of the matrix across the IM leading to a proton gradient used by ATP synthases to generate ATP within the matrix (Fig. 1B). From the matrix, ATP is transported into the intermembrane space (IMS) by the ATP/ADP translocator (ANT) in exchange to an ADP or ATP molecule. To reach the cytosol, ATP molecules have to cross the OM through voltage-dependent anion channels (VDACs). The complexes of the ETC and ATP synthases are mostly located at *cristae*, the invaginations of the inner membrane. These infoldings create specific compartments: the intercristal space (ICS), the narrower intermembrane space, and the internal matrix compartment (Fig. 1C).

The concrete morphology of mitochondria exhibits a large heterogeneity dependent on metabolic conditions^8,9^ and is associated with distinct physiological states and their specific subcellular energy demands^10^. Within the brain, mitochondria typically exhibit a composition of lamellar and tubular cristae^11^ and synaptic mitochondria, in particular, are further specialized to their physiological context by their smaller volume^11^, higher ratio of cristae to outer membrane surface^12^ and distinct metabolic profiles^5^.

While extended literature^6,10,13,14^ suggests a link between the inner membrane morphology and mitochondrial function, a mechanistic understanding is still lacking. This gap is caused by the small dimension and intricate structure of the mitochondrial ultrastructure which can be only resolved by electron tomography and leads to static and low throughput data. Investigating causal consequences of morphology on mitochondrial dynamics and function rely therefore to a large extent on computational modeling that allow for characterizing the effect spatial metabolic coupling has on the organelle behavior. Previous simulations of the interplay between morphology and the electrochemical potential predicted an increased proton concentration in the ICS compared to the IMS^15^. Effects on diffusion due to the internal structure that could effect energy metabolism were studied based on simplified geometries^16,17^ and indicated anomalous diffusion in some conditions^18^, but disagreed on the impact of the mitochondrial ultrastructure^16,17^.

**Figure 2:**
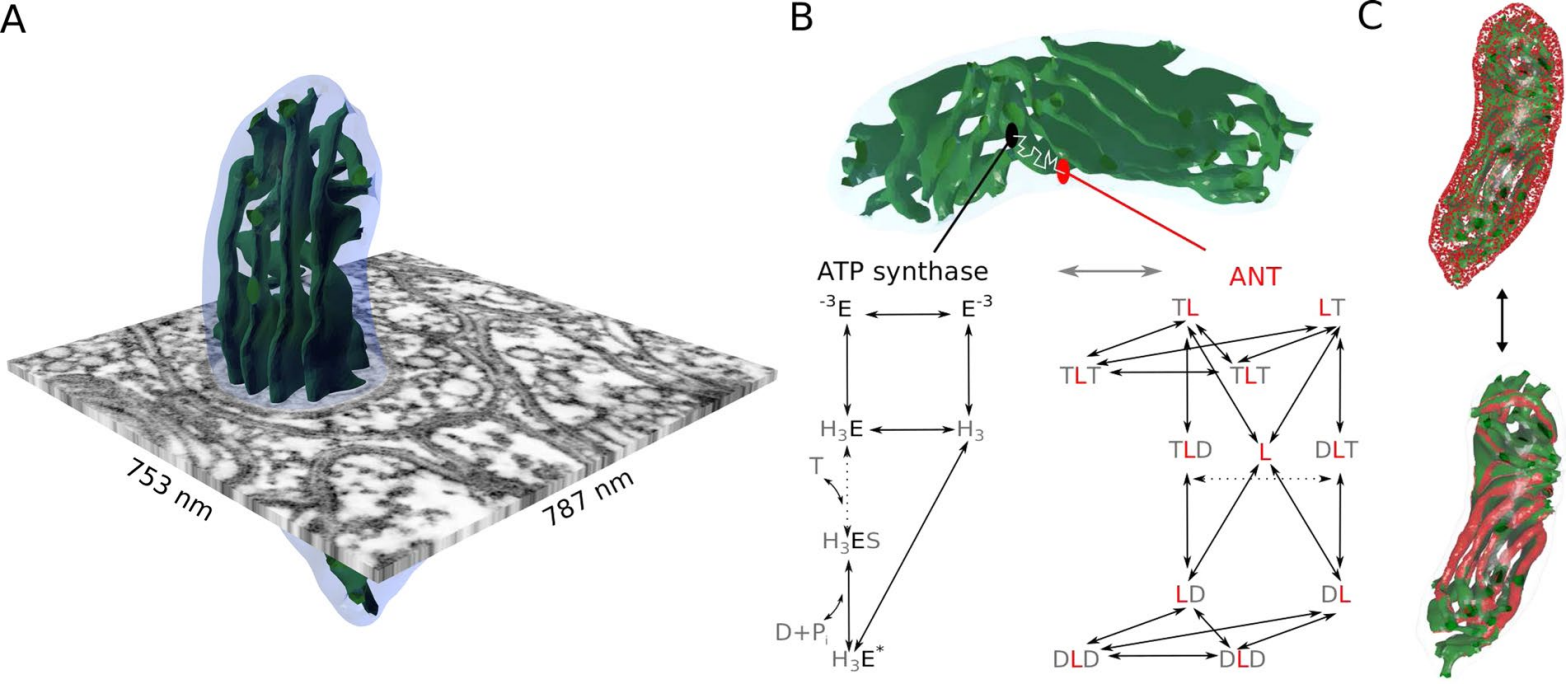
A physiological multiscale model of a mitochondrion based on electron microscopy tomography and dynamic simulations using MCell. (**A**) A presynaptic mitochondrion reconstructed from a serial electron-tomogram of a cerebellum mouse neuron where the synapse was embedded in a cube of 787 nm $\times$ 753 nm $\times$ 731 nm. (**B**) The mitochondrial multiscale model integrates the concrete morphology with Markov state models of the ATP (T) and ADP (D) handling proteins, the ATP synthase (black E) and the ANT (red L). The molecular dynamics considers the discrete binding of ATP and ADP within the matrix and the outside compartment and transitions also depend on protons (${{\rm{H}}}_{3}$) and phosphate (P${}_{i}$) availability (Methods). For the ANT, binding on the matrix site corresponds to the right letter of the protein L and binding from the outside to the left letter. The essential ATP generating step of the synthase and the ATP-ADP exchanging step of the ANT are indicated by the dashed arrows, respectively. (**C**) To investigate the dynamic effect of morphology in our simulations, ANTs were either homogeneously distributed on the IBM (top), co-localized with ATP synthases at the apex of cristae (bottom), or in both locations (not shown).

Since the interplay of diffusion with active molecules like transporters, proton pumps and synthases may have strong implications for the emergent dynamics dependent on spatial arrangement^19^, we developed a more realistic spatiotemporal mitochondrial model (Fig. 2) to (i) measure diffusion properties in a concrete physiological geometry, (ii) investigate how the interplay between diffusion and spatial localization of ANT and ATP synthase affect mitochondrial ATP production, and (iii) analyze potential energetic consequences for synaptic transmission. For this systematic investigation, our three-dimensional model is based on realistic morphologies reconstructed from electron microscopy tomograms (Fig. 2A) and uses Markov state transition models to describe the molecular dynamics of the adenosine processing proteins: ANT, ATP synthase, and VDAC (Fig. 2B). We used the static reconstructed geometry for multiscale simulations of the molecular interplay with spatially distinct molecular arrangements (Fig. 2C). The model was implemented in MCell^20,21^, an agent based reaction-diffusion simulator, and compared with a corresponding space-independent ordinary differential equation (ODE) approach.

We applied our model to a synaptic mitochondrion to analyze how brain specific mitochondrial morphology affects ATP production capacity. Interestingly, we found that morphology has only minor effects when the system relaxes towards an equilibrium steady state condition but spatial effects are amplified in non-equilibrium situations and may provide an energy buffering mechanism in more physiologically relevant conditions of a highly dynamic environment like the synapse^22,23^.

## Results

To investigate the effect of mitochondrial morphology on the ATP production, we systematically simulated different scenarios particularly for a synaptic mitochondrion with specific energy providing requirements. For this purpose, we reconstructed the morphology of an entire mitochondrion with unmatched precision and developed a multiscale model which considers specific physiological morphologies and the molecular dynamics of adenosine handling proteins (Fig. 2).

### Mitochondrial morphology reconstruction

Due to technical reasons, three-dimensional reconstructions of whole mitochondria are rare and accurate volume and surface measurements of mitochondria are lacking. We therefore initially focused on the comprehensive reconstruction of a synaptic mitochondrion from a serial electron tomogram volume (Fig. 2A). The resulting reconstruction was subsequently optimized to enable dynamic simulations and detailed morphological characterization (Supplementary Table 1) including the volume of 0.04 $\mu {m}^{3}$ with a maximal length of 0.8 $\mu$m and width of 0.29 $\mu$m and 45 cristae. Based on the physiological classification of mitochondria (Fig. 1C), we determined the size of the different compartments where the IMS occupies approximately a relative volume to the outer membrane of 0.27, the matrix 0.52 and the ICS 0.21.

### Isolated scenario of equilibration

**Figure 3:**
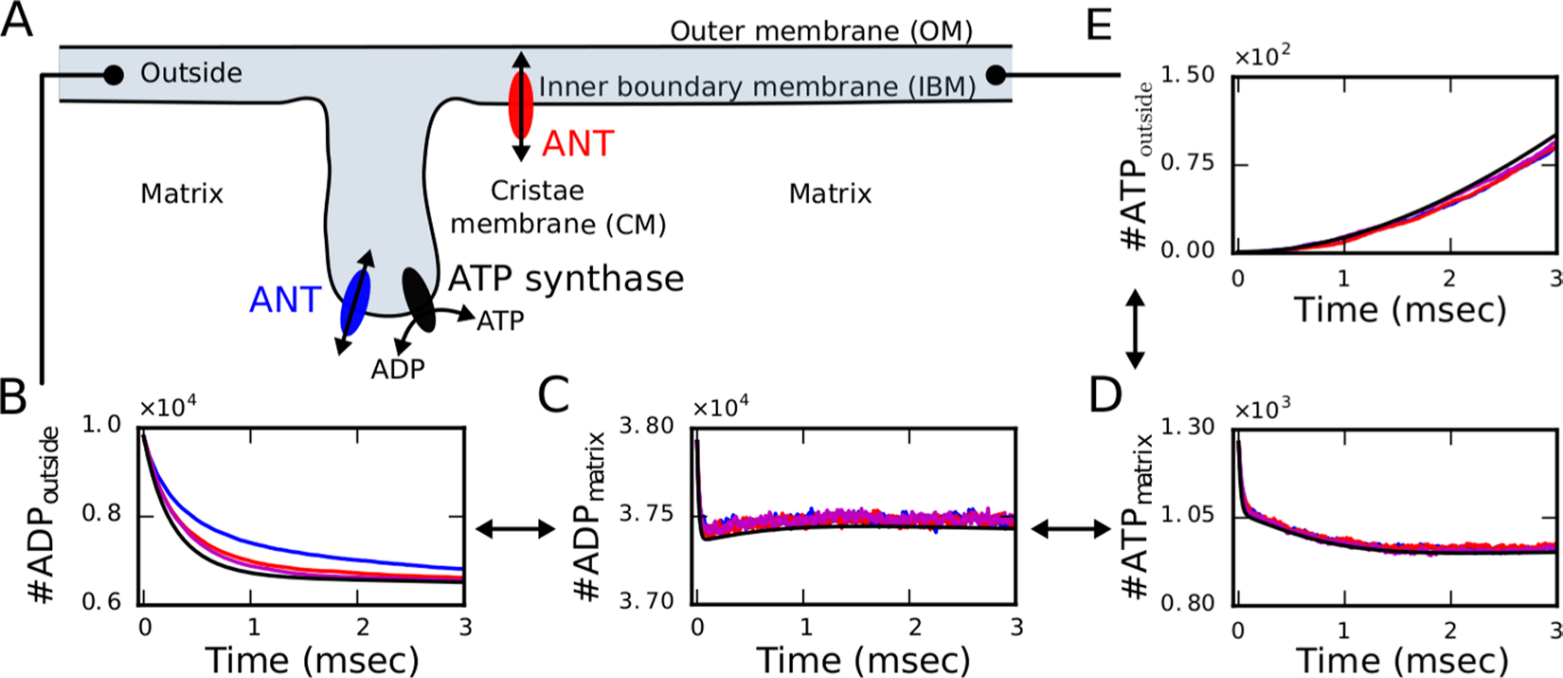
The isolated scenario of dynamic equilibration does not exhibit strong dependence on the spatial arrangement. (**A**) Schematic representation of the considered components and their arrangement, with ANTs either co-localized with ATP synthases in the cristae (blue), placed exclusively at the IBM (red) or in both locations. (**B**–**E**) Individual multiscale simulations are averaged over 10 different initial conditions for the distinct configurations of ANTs localized exclusively in the IBM (red), exclusively in the cristae (blue) or at both locations (magenta) and do not exhibit substantial differences compared to the corresponding ODE system (black). Among the spatial configurations, only the number of ADP molecules outside exhibited significant differences. The initially fixed number of ADP molecules in the outside compartment, which correspond to a concentration of 900 ${\rm{\mu }}$m, decreases (**B**) due to their import into the matrix (**C**) and is subsequently phosphorylated to ATP (**D**). From the matrix, ATP is exported into the outside where it accumulates over time (**E**) with no significant differences between the different arrangements of ANTs. (Statistical significance was assessed by Wilcoxon rank sum test with Holm compensation for multiple testing and significance levels p < 0.05).

To investigate the effect of the morphology on mitochondrial dynamics, we first considered a minimal configuration and simulated only the interplay between ANT and ATP synthase dependent on their spatial arrangements (Fig. 3A). In this scenario, ADP molecules corresponding to a free ADP concentration of 900 $\mu$M in the IMS and ICS (referred together as outside) are imported into the matrix by 20,000 ANT molecules and subsequently phosporylated to ATP by 3800 ATP synthases. The generated ATP can be eventually exported into cristae and the IMS by ANTs (Fig. 3B–E).

We focus here on the main readout of ATP and ADP molecules in the matrix and the outside space while the remaining variables are shown in Supplementary Fig. 3. During the equilibration process, we only observe minor differences between the different spatial arrangements within the first milliseconds which are caused by diffusion-induced delays (Fig. 3B–E). Interestingly, differences in the spatially independent ODE system (black lines) are more pronounced when ANTs are co-localized with ATP synthases at the apex of the cristae (blue) because ADP molecules in the outside first have to diffuse within the cristae to be subsequently imported into the matrix by ANTs located in the CM (Fig. 3B). Nevertheless, these differences are rather small and specifically the exported ATP does neither exhibit a significant dependence on morphology nor on the molecular spatial arrangement.

### Non-Equilibrium induced gradients

**Figure 4:**
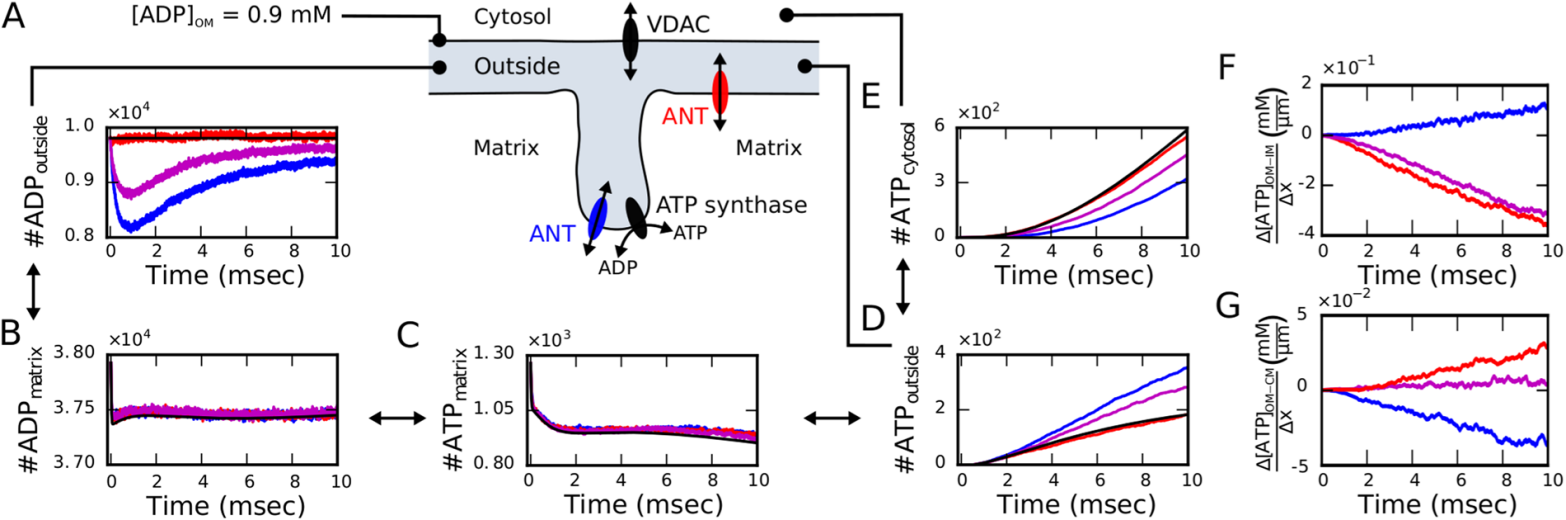
Non-equilibrium dynamics of the synaptic mitochondrion driven by clamped ADP concentration and ATP export. (**A**) Schematic representation of the components included in these simulations and dynamics of ADP molecules in the outside compartment. (**B**–**E**) The comparison of averaged molecule trajectories for the distinct ANT localizations (ANTs homogeneously distributed in the IBM in red; ANTs colocalized with ATP synthases at the most curved region of the CM in blue; ANTs in both locations in magenta) with results of the ODE system (in black) exhibits substantial spatial effects for exported ATP in case of colocalization (blue). (**F**–**G**) These differences are driven by diffusion-limitation induced sub-organelle ATP gradients between the outer and inner membranes (**F**) and between the outer and cristae membranes (**G**), respectively. For clarity, data were smoothed with a moving window of 500 data points. Among the spatial configurations, the number of molecules of ADP outside, ATP outside and ATP in the cytosol were significant different as assessed by Wilcoxon rank sum test with Holm compensation for multiple testing and significance levels p < 0.05).

To investigate the mitochondrial dynamics under a more physiological non-equilibrium condition, we clamped the concentration of ADP at the surface of the OM to 900 $\mu$M mimicking unlimited ADP resources in the cytosol, and included VDACs in the OM (Fig. 4A) to export ATP into the cytosol. For this extended model, we monitored again the main variables of the system including the amount of exported ATP in dependence on the different spatial arrangements and compared averaged trajectories with the corresponding ODE system (Fig. 4A–E and Supplementary Fig. 4).

In this driven system, different ANT configurations lead to distinct dynamics. When ANTs are distributed in the inner boundary membrane (IBM, red), the outside ADP concentration is almost constant but for ANTs located in the cristae membrane (CM, blue) an initial drop in the ADP concentration is caused due to a local depletion of ADP in the ICS (Fig. 4A). Initially, all ADP molecules are homogeneously distributed in the outside space consisting of IMS and ICS. If ANTs are located in the IBM (red), ADP molecules are quickly bound to free ANT proteins but ADP molecules are immediately replenished from the clamped membrane concentration. Hence, no local gradients are formed. If ANTs are located in the CM exclusively (blue), ADP molecules in the ICS are quickly bound to free ANT proteins leading to a decrease of the ADP concentration in the cristae volume. This induced concentration gradient transitorily attracts more molecules from the IMS. Since the replenishment relies on slow diffusion through tubular cristae junctions (CJs) of small diameters ($\sim$25.5 nm in our reconstructed mesh) connecting the cristae with the peripheral volume, the drop in the outside ADP is enhanced in amplitude as well as in duration. To further characterize this scenario, we estimated the concentration dynamics in the IMS and the ICS (Supplementary Fig. 4) and found that the initially induced ADP gradient is reducing over time and represents the driving force for the persistent differences in the outside ADP between the different configurations (Fig. 4A).

The differences in the outside ADP concentrations are accompanied with differences in the outside ATP concentration (Fig. 4D) where more ATP is present in the outside if ANTs are distributed in the CM (blue). In this configuration, ATP molecules are exported into the cristae volume from where they first have to diffuse into the IMS to react with VDACs in the OM for export from the mitochondrion. This diffusive transport takes longer compared to the scenario when ATP is directly exported to the peripheral volume (e.g. when ANTs are located in the IBM, red). Therefore, when ANTs are in the CM, more ATP molecules are found in the outside space because they are more persistent in the ICS. To understand this interplay in more detail, we estimated the trajectories of ATP concentrations in the IMS and ICS (Supplementary Fig. 1) and quantified the resulting gradients (Fig. 4F,G and Supplementary Section S1 with Supplementary Fig. 2). The larger and negative ATP gradients between the OM and IBM when ANTs are located in the IBM (red) facilitate ATP transport towards the cytosol (Fig. 4E) and deliver approximately double the ATP amount compared to ANTs located in the CM. Remarkably, in this non-equilibrium scenario, the setup with ANTs in the IBM does not exhibit any major differences to the space-independent ODE model whereas localization of ANTs in the cristae induces diffusion limitation for cytosolic ATP export.

### Morphologically buffered energy production at a presynaptic terminal

**Figure 5:**
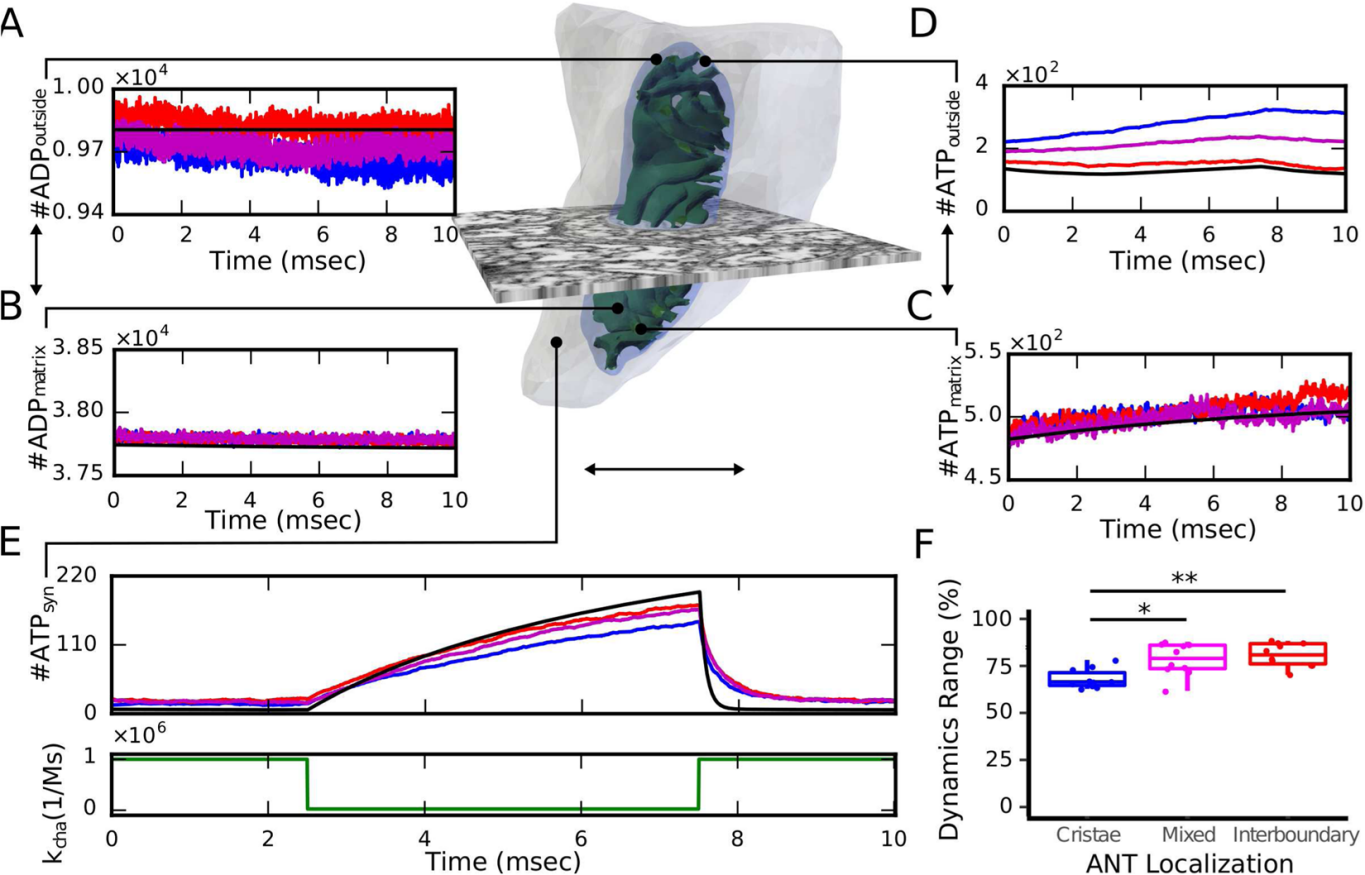
The energy dynamics at a presynaptic terminal. (**A**) In this configuration, the mitochondrion is placed within the segmented synapse (grey surface) and we considered ATP-consuming reactions. (**B**–**E**) Comparison of averaged molecule trajectories in the different compartments (color) and corresponding ODE results (black) for simulations of an action potential arrival at the presynaptic terminal by modulating the rate constant ${k}_{{\rm{cha}}}$ of the ATP-consuming reactions. As before, the concentration of ADP in the OM was clamped and VDACs included in the OM. As before the number of ATP molecules outside exhibited significant differences between the conditions (p < 0.05). ANT molecules were placed in three different locations: ANTs homogeneously distributed in the IBM (red), ANTs colocalized with ATP synthase at the most curved region of the CM (blue), and ANTs in both locations (magenta). (**F**) The dynamic range of the synaptic ATP is significantly reduced in multiscale simulations compared to the space independent ODE model. For each of the 10 individual simulations per condition, the dynamic range was determined by the difference between the maximum and the basal number of ATP molecules and related to the ODE model. ANT localization at the cristae (blue) exhibited a significant reduced dynamic range compared to localization in the IBM (red) and the mixed condition (magenta). (Statistical significance was assessed by Wilcoxon rank sum test with Holm compensation for multiple testing and significance levels *p < 0.05: and **p < 0.01).

After model establishment and finding significant differences in the cytosolic ATP production in dependence on the spatial arrangement, we were interested in potential physiological consequences of morphology on the synaptic dynamics. For this purpose, we investigated the ATP production rate of the mitochondrion in its physiological context, the presynaptic terminal (Fig. 5A), and included ATP-consuming reactions at the synaptic membrane to emulate the arrival of an action potential at the terminal by varying the rate constant ${k}_{{\rm{cha}}}$ of the ATP-consuming reactions. Based on estimations of the energetic costs of a glutamatergic synapse, we set the basal ATP consumption rate to ${k}_{{\rm{cha}}}=2.5\cdot 1{0}^{4}$ (Ms)${}^{-1}$ and the energy demand during an action potential to ${k}_{{\rm{cha}}}=1\cdot 1{0}^{6}$ (Ms)${}^{-1}$ (Supplementary Section S2 for parameter estimation). To study how synaptic activation induces a transient transition between the approximated steady states for the different scenarios (Fig. 5A–E), we simulated the energetic response during a 5 milliseconds lasting recovery phase between 2 spikes by modulating ${k}_{{\rm{cha}}}$ as step functions between the basal and active ATP consumption rates (Fig. 5E).

In contrast to the previous scenario, these simulations started close to steady state conditions determined by the ODE system. Therefore, we did not observe an initial dip in the outside ADP concentration (Fig. 5A) but a stable gradient that drives the differences among the distinct configurations. Due to ADP clamping, the outside ADP concentration stays constant for the ODE approach and similarly for the scenario where ANTs are localized in the IBM (black and red in Fig. 5A, respectively), whereas for ANTs exclusively or partly localized in the cristae, a small drop is observed (blue and magenta in Fig. 5A, respectively). Interestingly, ADP as well as ATP concentrations in the matrix are at least slightly increased consistently for all spatial simulation compared to the ODE approach (Fig. 5B–D).

The most predominant difference is subsequently observed in the outside ATP concentration where localization of ANTs in the IBM again exhibit similar concentrations as the ODE system whereas localization of ANTs in the cristae lead to substantially increased ATP levels (red and blue in Fig. 5D, respectively). As in the non-equilibrium scenario, this increase is caused by ATP within the ICS from where it first has to diffuse to the IMS for subsequent export into the cytosol. Hence, the cytosolic ATP is slightly lower for ANTs located in the cristae compared to ANTs in the IBM (blue and red in Fig. 5E). Despite this difference, all spatiotemporal scenarios exhibited consistently smaller ATP amounts within the synapse during the recovery period with low energy demand compared to the ODE simulations (24% less for co-localization vs 10% less for IBM localization). After synaptic activation, the spatiotemporal simulations displayed a slower decrease in synaptic ATP and reduced differences in the ATP concentration between base level and activation conditions.

To quantify the effect of this different dynamics and assess the statistical significance, we determined the dynamic range of each individual simulation by the difference between the maximal and basal number of ATP molecules in relation to the ODE results (Fig. 5F). We find that the arrangement of ANTs in the cristae exhibits a significantly reduced dynamic range of 70% compared to the space independent ODE approach. Interestingly, the ANT arrangement in the interboundary membrane exhibits a similar reduced dynamic range as the mixed arrangement of 85% compared to the ODE system, which are significantly higher than for the cristae arrangement. These results together indicate that ATP molecules can be buffered by the complex morphology and support adaptation to variable conditions.

We subsequently used the presynaptic model to calculate net ATP production rates from the peak in Fig. 5E. For ANTs located in the IBM we calculate a rate of $\sim$31 molecules/ms slightly reduced compared to the ODE system ($\sim$38 molecules/ms). The model with ANTs exclusively in the CM exhibits a rate of $\sim$26 molecules/ms. Comparison with theoretical estimations (Table 1) and approximations in the literature exhibit good agreement.

### Dependence on the mitochondrial membrane potential

**Figure 6:**
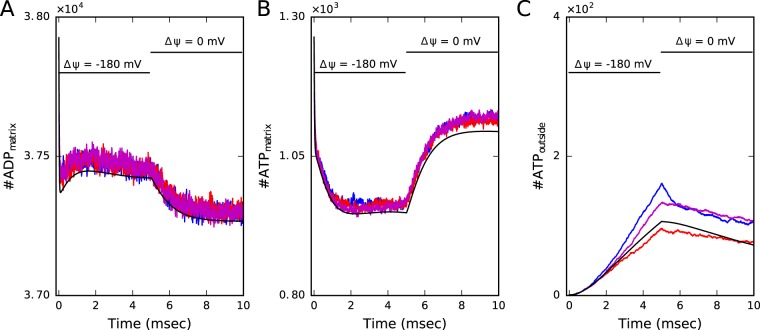
Dependence on the mitochondrial membrane potential. In this configuration we start our simulations with a functional mitochondrion in the same scenario as in Fig. 4 and at 5 milliseconds we emulate the breakdown of the membrane potential from $\Delta \psi =-\,180$ mV to $\Delta \psi =0$ mV by changing the corresponding protein activity rates (Supplementary Tables 2 and 3). Under these conditions ATP synthases work stochastically in a reversible form hydrolyzing and phosporylating ATP, leading on average to a reduction in the number of ADP in the matrix (**A**) and to an increase in the number of ATP in the matrix (**B**). Overall, under these conditions the capacity of the organelle to produce ATP is abolished and no more ATP molecules reach the outside (**C**).

The mitochondrial membrane potential is an essential mechanism of the organelle function and its stressed-induced breakdown is associated with mitophagy and several diseases. To investigate the potential interaction of the morphology with the membrane potential, we simulated a scenario where the membrane potential is completely abolished by changing the corresponding protein activity rates (see Supplementary Tables 2 and 3 for details of parameter values). We start from the same configurations presented in the non-equilibrium scenario (Fig. 4) and at 5 milliseconds we set the membrane potential to zero (Fig. 6A–C). Under these conditions, ATP synthases work stochastically hydrolyzing and phosphorylating ATP, what produces on averaged a reduction in the number of ADP in the matrix (Fig. 6A). Furthermore, ANTs are not efficiently exporting ATP, producing an increment in the number of ATP in the matrix (Fig. 6B). Overall, we found that under these conditions the capacity of the organelle to produce ATP is suppressed, and no more ATP molecules reach the IMS (Fig. 6C). Interestingly, the buffering effect is also found in this scenario, and differences between the spatial configurations are observed.

## Discussion

High-resolution, 3D reconstructions of mitochondria can be obtained from electron tomography^24^ with typical resolutions between 3 and 20 nm, depending on several factors, including sample preparation, section thickness and electron microscope voltage. For the best ultrastructural preservation, electron tomography samples should be either frozen-hydrated^25^ or high-pressure frozen followed by freeze-substitution with fixatives (HPF-FS)^26^. While frozen-hydrated tissue samples may be conserved in close-to-physiological conditions, they are notoriously difficult to section and even when sections are obtained, they often suffer from micro-crevasses, pits and material lost between cuts. Thus, HPF-FS remains the technique of choice for producing a large volume of a well-preserved tissue that will subsequently be sectioned with minimal material loss. Semi-thick to thick-section samples are typically in the range of 200-3000 nm, with the thicker sections suitable for high-voltage electron microscopes. With this approach, a significant fraction of a mitochondrion can be embedded within the sample, but due to the voltage of 300 kV used, with most types of mitochondria, it is rather unlikely to include an entire mitochondrion within the section. Given the aforementioned technical reasons, 3D reconstructions of entire, well-preserved mitochondria are rare and analyses from the literature are typically based on 2D images or partial 3D reconstructions. Therefore, serial tomography was used to preserve the high-resolution afforded by semi-thick sections by stacking 3 serial volumes together to generate a final volume large enough to include a whole synapse with a nearby complete mitochondrion for a comprehensive modeling study. Based on the resulting *in silico* representation, we performed precise measurements in the reconstructed volume as detailed in Table 1 of the Supplementary Material. Previously reported distances between the OM and the IM in brain mitochondria exhibiting a combination of lamellar and tubular cristae^11^ are similar to our results, whereas the determined values for the diameter of crista junctions were slightly smaller ($\sim$16 nm) compared to our measurements ($\sim$ 25 nm). The estimated crista junction density of $\sim 83\mu {m}^{-2}$ in our sample is smaller than previous estimations^12^ of $\sim \,136\,\mu {m}^{-2}$. The volume and surface membrane of mitochondria in liver^27^ exhibit larger values compared to the here analyzed mitochondrion in agreement with a previous report on the small volume of synaptic mitochondria^11^.

**Table 1:** Estimation of ATP production in synaptic mitochondria. For details in the calculations refer to the supplementary material.

#ATPs/s	Reference
0.56 x 10${}^{5}$	MCell simulations (ANTs in the CM)
0.72 x 10${}^{5}$	MCell simulations (ANTs in the IBM)
0.95 x 10${}^{5}$	ODE simulations
4.60 x 10${}^{5}$	theoretical estimation
7.00 x 10${}^{5}$	^22^
6.02 x 10${}^{5}$	^23^

Mitochondrial morphology is thought to be context dependent and a mechanism to adapt to specific energetic requirements^13,14^. Mitochondria in the brain and specifically at synapses exhibit rather unique and complex morphologies^11,12^ that may reflect the high energy demand for neuronal information transmission^22^. Since the internal structure of mitochondria can be only resolved by electron tomography, a mechanistic understanding of how morphology is affecting mitochondrial dynamics relies on mathematical modeling to simulate dynamic consequences from the static images.

While modeling approaches have estimated the morphological effect on the mitochondrial membrane potential^15^ and diffusion properties based on simplified geometries^17,18^, the consequences for the main function of ATP production of a real physiological morphology is only vaguely understood. Here, we used an electron tomogram of a presynaptic terminal in mouse cerebellum to (i) comprehensively reconstruct and analyze in detail the morphology of an entire mitochondrion (Supplementary Table 1) and to (ii) subsequently investigate the dynamic consequences of the interplay between the complex morphology the spatial molecular orchestration by our developed computational model based on the mitochondrial morphology and molecular properties of the main adenosine phosphate processing molecules.

Surprisingly, simulations of the isolated scenario without any ADP import from and ATP export into the cytosol do not exhibit a strong dependence on the spatial arrangement (Fig. 3) indicating that the assumed diffusion properties do not lead to a diffusion limiting condition. In accordance with theoretical considerations, comparing the timescales of diffusion and reactions indicated only a slight overlap for this scenario (Supplementary Section S2). A morphological effect on ATP production could only be found for diffusion coefficients decreased by two orders of magnitude (Supplementary Section S1). Although some studies^13,17,28^ showed evidence of severe hindrance of diffusion in the matrix, other experiments estimated that diffusion is only three to four fold smaller than in water^16^. In our model, we reduced the diffusion coefficient of ATP and ADP by one order of magnitude to reflect their ionized form and related interactions with other charged particles. The potential interaction of the ions with the membrane potential leading to electro-diffusion are not included in the current model but could actually decrease diffusion further and induce a regime of diffusion limitation. Independent of the diffusion limitation, our simulations indicated anomalous diffusion in agreement with previous evidences^18^.

Although diffusion had only a minor effect in the isolated system, spatial aspects became significant when bringing the mitochondrion in contact with the cytosol under unlimited access to ADP and ATP export through VDAC (Fig. 4). Under these more physiological conditions, the spatial organization of ANTs had a significant effect on ATP gain within the cytosol. While the spatiotemporal simulations did not exhibit significantly strong deviations from the spatially independent ODE system when ANTs were exclusively located at the IBM, the co-localization of ANTs with ATP synthases at the apex of cristae led to an approximately halved ATP export into the cytosol. Careful analysis of the dynamics revealed that this effect is driven by smaller concentration gradients between the ICS and the OM for ANT localization in the cristae, which led to ATP buffering within the cristae. This scenario is in contrast with the greater concentration gradient formed between the IBM and the OM when ANTs are located in the IBM what is facilitating ATP transport into the cytosol. These findings quantitatively support the importance of sub-organelle gradients suggested in the literature^29^.

To test whether this buffering mechanism might have an effect on synaptic physiology and explain the distinct morphology of brain and specifically of synaptic mitochondria, we subsequently simulated the mitochondrion in its synaptic environment with a variable cytosolic ATP consumption reflecting changes during synaptic transmission. These simulations have shown that ATP buffering in cristae caused by the non-equilibrium induced gradients is a mechanism to buffer large energy demand peaks.

We finally used our detailed model to calculate the ATP production rate of the considered mitochondrion for the different scenarios. The resulting rates of $\sim$10${}^{5}$ molecules of ATP per second are in agreement with our theoretical estimation based on the ANT translocation rate and the ANT density in mitochondria (Supplementary Section S2). These values are further supported by independent approximations found in the literature^22,23^ (Table 1) where minor deviations to previous estimations^22^ would rematch for firing rates of 30 Hz. The main mechanism how mitochondria decode the firing rate is probably Ca${}^{2+}$ influx through the mitochondrial calcium uniporter (MCU)^30^. Incorporating the MCU and the effect of Ca${}^{2+}$ on the membrane potential in a future version of the model will allow for more detailed predictions of ATP production rates in dependence on neuronal activity.

Morphological variability has been found in mitochondria of different tissues and cell types, not only in size but also in their ultrastructure^11^. Moreover, enzymatic differences have been reported with respect to the content and activity of mitochondrial proteins. For instance, ANTs in liver cells exhibit higher turnover rates than in synaptic mitochondria^31^, and the current through the mitochondrial calcium uniporter is lower in the heart than in liver^32^. These differences are often accompanied by tissue specific protein concentrations^32^. While our results are based on one specific synaptic mitochondrion, the model we have established here can be easily adapted to other morphologies or enzyme concentrations and localizations. Thereby, tissue or cell-specific mitochondrial morphologies and enzyme densities will lead to distinct ATP production rates and buffering effects where e.g. mitochondria exclusively formed by lamellar cristae, as in brown fat tissue, will exhibit an increased buffering capacity. Based on the here developed methodology, a systematic investigation of cell-type and tissue-specific mitochondrial morphologies and enzyme abundances will allow further characterization of mitochondrial specialization in different conditions such as stress^33^, aging or sex-related hormones^34^.

Overall, our systematic approach with our detailed mitochondrial model has shown that the concrete morphology of the presynaptic mitochondrion induces anomalous diffusion but has not per se an impact on ATP production when the system relaxes towards an equilibrium steady state (Fig. 3). In contrast, the spatial arrangement of ANTs under non-equilibrium conditions induce sub-organelle gradients that led to a significant effect on the cytosolic ATP concentration (Fig. 4F). Physiological simulations of the synaptic dynamics suggest that this buffering effect might be a mechanism to smear out the variable energy demands (Fig. 5) and may therefore increase robustness and adaptability of synapses and explain the distinct morphology of brain mitochondria.

## Methods

Spatiotemporal simulations were performed with MCell (version 3.4)^21^ and compared with space-independent simulations of the corresponding rate equation system. For the spatiotemporal model, each molecular component was first implemented independently, parameterized and validated by experimental data and eventually combined in the realistic mitochondrial model. The entire dynamical system has 21 variables (6 for ATP synthase, 11 for ANT and 4 for ADP and ATP concentrations within the 2 compartments).

### Specimen preparation

A 1-month old C57BL/6NHsd male mouse was anesthetized with ketamine/xylazine and transcardially perfused with Ringer’s solution followed by 2.5% glutaraldehyde, 2% formaldehyde, 2 mM ${{\rm{CaCl}}}_{2}$ in 0.15 M sodium cacodylate buffer. The fixation was started at 37 °C and the fixative was cooled on ice during perfusion. The brain was post-fixed after removal from the cranium in the same fixative solution for 1 hour at 4 °C. The cerebellar vermis was cut into 100 $\mu$m thick sagittal slices on a vibrating microtome in ice-cold 0.15 M cacodylate buffer containing 2 mM ${{\rm{CaCl}}}_{2}$ and briefly stored in same buffer prior to high pressure freezing (HPF). A 1.2 mm tissue punch was taken from a tissue slice and placed into a 100 $\mu$m deep membrane carrier filled with 20% bovine serum albumin in cacodylate buffer and frozen with an EM PACT2 HPF apparatus. The specimen was freeze substituted in extra dry acetone (Acros) using an AFS2 as follows: 0.1% tannic acid at −90 °C for 24 hours, wash 3x 20 min in acetone, 2% ${{\rm{OsO}}}_{4}$/0.1% uranyl acetate at −90 °C for 48 hours, warmed for 15 hours to −60 °C, held at −60 °C for 10 hours, and warmed to 0 °C over 16 hours. The specimen was infiltrated with a series of Durcupan ACM: acetone solutions and then embedded in 100% Durcupan at 60 °C for 48 hours. All animal procedures were Institutional Animal Care and Use Committees at the University of California, San Diego (USA).

### Electron tomography

300 nm sections were cut and collected on 50 nm thick Luxel slot grids. The sections were glow discharged and coated with 10 nm colloidal gold. Tilt series were collected on an FEI Titan 300 kV microscope with a 4k x 4k CCD detector (Gatan Ultrascan). Four tilt series were collected from the region of interest at 0, 45, 90, and 135 degrees rotation of the specimen plane. Each tilt series was collected from −60 to +60 degrees with 1${}^{\circ }$ increments. Projection images were collected with a pixel size of 0.4 nm, and images were binned by 4 prior to tomographic reconstruction with TxBR^35^. The serial electron tomogram is shown in Supplementary Movie 1.

### Model geometry

Mitochondrial and synaptic three-dimensional *in silico* reconstructions were performed from 3 sections of a serial electron tomogram of a high pressure frozen/freeze substituted^26^ cerebellum sample, exhibiting final pixel resolution of 1.64 nm, leading to a stack of 360 images containing the mitochondrion and the synapse. First, membranes of the presynaptic mitochondrion were manually traced using RECONSTRUCT. Afterwards, contours were converted into three-dimensional surfaces by VolRover. Finally, meshes were imported into Blender to generate a triangulated, watertight and manifold mesh using CellBlender’s Mesh Analysis tool. Further optimization was performed with the mesh improvement library and Blender add on GAMer. To consider possible compression effects vesicles were traced, and its shape was set to spheres of diameter 40 nm. We found shrinkage in the Z direction of 20%, in order to correct for this we rescaled the reconstructed meshes by a factor of 1.239 in the Z direction. Supplementary Movies 2 and 3 visualize the complex morphology (Supplementary Section S1).

### Molecular ATP/ADP translocator (ANT) model

The ANT model is based on the work of Metelkin *et al*.^36^. Two additional states were added to track futile translocations in MCell. The resulting kinetic ANT model (Fig. 2B) is composed of 11 states and 19 bidirectional transitions between them resembling the binding and unbinding of ATP and ADP from different sides of the IM. Starting from fitted flux parameters^36^ for ANT extracted from heart mitochondria^37^, we first estimated parameters for the implementation in MCell and the corresponding ODE model (Supplementary Section S2). With this set of parameters, we qualitatively reproduced the independent data from published work^37,38^. To obtain a reference ATP turnover rate, we used published data for synaptic mitochondria^31^. The complete list of parameters are given in the Supplementary Table 2. The location of ANTs in mitochondria has not yet been definitively determined. Experimental evidence show on the one hand that they may form complexes with ATP synthases and phosphate carriers^39^ located in the CM^40,41^ and, on the other hand, studies report an association with VDACs located in the IBM^42^. In our simulations we explored the functional implications of these different locations by placing them (i) homogeneously distributed in the IBM (Fig. 2C, top), (ii) colocalized with ATP synthases in the CM (Fig. 2C bottom) or (iii) in both locations.

### Molecular ATP synthase model

The ATP synthase model is based on the six state model of a proton pump by Pietrobon and Caplan^43^ shown in Fig. 2B. A clockwise cycle starting in ${E}^{-3}$ represents the binding of 3 protons from the IMS, transport of the protons, binding of ADP and phosphate (P${}_{i}$) and subsequent synthesis of ATP, followed by unbinding of the protons in the matrix. In our model, we considered the proton concentration inside the ICS as well as proton and phosphate concentrations in the matrix to be constant and used ADP and ATP in the matrix and the IMS as input variables. In our model, ATP synthases were localized at the apex of the CM in lamellar cristae and along the length of tubular cristae, in accordance to experimental findings^44^. All model parameters are given in the Supplementary Table 3.

### Molecular VDAC model

To consider processes that export ATP from the mitochondrion into the cytosol, we included VDACs, the main mechanism for metabolites to cross the OM. We implemented a rather basic model of VDAC assuming that VDAC proteins interact with ATP and translocate it to the cytosol by the reaction ${\rm{V}}{\rm{DAC}}+{{\rm{ATP}}}_{{\rm{mito}}}\rightleftharpoons {\rm{V}}{\rm{DAC}}+{{\rm{ATP}}}_{{\rm{cyto}}}$. In our simulations VDAC proteins were homogeneously distributed within the OM with a density of $1{0}^{4}\mu {m}^{-2}$ (ref. ^45^ and Supplementary Section 2 and Table S5 for details and parameters values).

### Metabolite diffusive properties and buffers

Diffusion coefficients were estimated previously based on measurements of green fluorescent protein (GFP) in the matrix of mitochondria of diverse cells^16,17^ reporting that the free diffusion is two to fourfold reduced compared to water^16,17^. For our simulations, the free diffusion coefficient is relevant since the effect of morphology is included in our model. Although GFP as a protein has a higher molecular weight than ATP or ADP and as such would have a lower diffusion coefficient, ATP and ADP are ionized in neutral solutions as ATP${}^{4-}$ and ADP${}^{3-}$ leading to lower mobility due to interactions with other charged particles and the electrochemical gradient at the membrane^46^. To account for these interactions, we reduced the free diffusion coefficient by one order of magnitude to $1.5\cdot 1{0}^{-7}{{\rm{cm}}}^{2}{{\rm{s}}}^{-1}$. Comparisons for lower diffusion coefficients are given in Supplementary Figs. 5, 6 and 7.

ADP and ATP can react with different cations, be bound or ionized. Therefore, the total concentration of ATP can be distributed in different compounds or states like ATP${}^{4-}$, ATPMg${}^{2-}$. These distributions can be estimated by coefficients representing the fraction of unbound ATP in the matrix of mitochondria or the external compartments. For our model, mitochondrial ADP${}^{3-}$ and ATP${}^{4-}$ concentrations were estimated analogously to published data^47^ as [ADP]${}_{{\rm{m}},{\rm{f}}{\rm{r}}{\rm{e}}{\rm{e}}}$ = 0.8 [ADP]${}_{{\rm{m}}}$, [ATP]${}_{{\rm{m,free}}}$=[ATP]${}_{{\rm{m}}}$, [ATP${}^{4-}$] = 0.05 [ATP] and [ADP${}^{-3}$] = 0.45 [ADP]${}_{{\rm{free}}}$. The concentrations of ATP and ADP in the matrix were set to 2 mM and 10 mM, respectively, and to 0.01 mM and 2 mM in the cytosol.

### Space-independent ODE approach

For each molecular model, we also developed a corresponding ODE approach describing the fluxes based on mass action kinetics (Supplementary Section S2). The ODEs were integrated by PyDSTool^48^. To investigate morphological effects, the different spatial configurations simulated with MCell were compared with corresponding solutions of the ODE system.

### Numerical experiments

For model establishment, we performed 3 distinct *in silico* experiments to disentangle the contribution of the different molecular components to the dynamics. In a first set of simulations, we started with a fixed number of ADP molecules and let them be phosphorylated to ATP without any export or consumption of ATP. Hence, in this *isolated scenario*, ATP molecules accumulate in the mitochondrion. In a second configuration, we consider the mitochondrion to be embedded in a cube of dimension 0.45 $\mu$m${}^{3}$ reflecting the cytosol with unlimited resources of ADP by clamping the concentration of ADP in the OM, and include VDAC in the OM for mitochondrial export. The more *physiological scenario* of a fluctuating energy demand at a synapse is similar to the scenario of unlimited resources but with the mitochondrion located in the reconstructed synapse. ATP-consuming reactions are included at the synaptic membrane representing different ATP-consuming processes. The activation of the reactions due to action potential arrivals was implemented by an increase in the rate constant of the ATP-consuming reactions. To ensure statistical significance of the morphology mediated effects, we ran for each condition 10 individual simulations with different realizations of concrete protein localization and initial ADP and ATP distributions. The shown time courses represent averaged trajectories and statistical significance between conditions was accessed by Wilcoxon rank sum test of end point measurements of individual trajectories with Holm compensation. This allowed for comparison between configurations and with the spatially independent scenario described by the corresponding ODE system.

### Ethics

All animal procedures were approved and followed the guidelines of the Institutional Animal Care and Use Committees at the University of California, San Diego (USA).

## Supplementary information

Supplementary Information

Supplementary Video

Supplementary Video

Supplementary Video
